# Protective Effects of *Smilax glabra* Roxb. Against Lead-Induced Renal Oxidative Stress, Inflammation and Apoptosis in Weaning Rats and HEK-293 Cells

**DOI:** 10.3389/fphar.2020.556248

**Published:** 2020-09-02

**Authors:** Yueyue Shi, Chongmei Tian, Xinfen Yu, Yuejuan Fang, Xinyu Zhao, Xiaoxi Zhang, Daozong Xia

**Affiliations:** ^1^ College of Pharmaceutical Sciences, Zhejiang Chinese Medical University, Hangzhou, China; ^2^ Department of Pharmacy, Affiliated Shaoxing Hospital of Traditional Chinese Medicine, Zhejiang Chinese Medical University, Shaoxing, China; ^3^ Center of Health Laboratory Technology, Hangzhou Center for Disease Control and Prevention, Hangzhou, China

**Keywords:** *Smilax glabra* Roxb., lead-induced nephrotoxicity, apoptosis, oxidative stress, inflammatory pathway

## Abstract

Lead (Pb) is an important environmental pollutant. Oxidative stress and the inflammatory response have been postulated as mechanisms involved in lead-induced renal damage. *Smilax glabra* Roxb. has been used for treatment of heavy-metal poisoning in China for 500 years. We investigated *S. glabra* flavonoids extract (SGF) could attenuate lead acetate-induced nephrotoxicity in weaning rats and human embryonic kidney (HEK)-293 cells, and investigated the possible mechanisms. Compared with Pb exposed group of weaning rats, SGF could significantly promote lead excretion in the blood and kidney, and increase the content of the renal-function indicators blood urea nitrogen, serum uric acid, and serum creatinine. SGF could improve the glomerular filtration rate (GFR) and histologic changes in the kidneys of weaning rats exposed to Pb. SGF could also reduce lead-induced cytotoxicity, improve DNA damage-induced apoptosis and cleaved caspase-3-mediated apoptosis in HEK-293 cells stimulated with Pb. SGF significantly increased the activity of the antioxidant enzymes superoxide dismutase, glutathione peroxidase and catalase, and decreased excessive release of reactive oxygen species (ROS) and malondialdehyde in the kidneys of the weaning rats and in HEK-293 cells. The antioxidant mechanism of SGF related to activation of the Kelch-like ECH-associated protein 1/nuclear-factor-E2-related factor 2/hemeoxygenase-1(Keap1/Nrf2/HO-1) pathway. SGF could inhibit secretion of interleukin (IL)-1β, IL-6 and tumor necrosis factor (TNF)-α induced by Pb *in vivo* and *in vitro*. The anti-inflammatory mechanism of SGF related to inhibition of ROS and pro-inflammatory cytokines triggered the nuclear factor-kappa B (NF-κB) pathway through blockade of inhibitors of I-κB degradation, phosphorylation of NF-κB p65, and nuclear translocation of p65. Our findings indicate that SGF could be a natural antioxidant and anti-inflammatory agent for treating lead-induced nephrotoxicity.

## Introduction

Lead (Pb) is an important environmental pollutant. It can enter the body through intake of contaminated food and water, as well as through the inhalation of polluted air or dust (Mohamed et al., 2020). With increased industrialization in China, lead poisoning has become an important health risk for people. It can have devastating effects not only on the neurodevelopment, but also on the physiologic functions of the kidney and other organs, in children (Xia et al., 2010; Gargouri et al., 2020). Studies on lead toxicology and neurodevelopment in children have suggested it can result in intellectual disability and a low intelligence quotient, which then leads to poor performance in school and behavioral disorders (Yabe et al., 2020). Moreover, the kidney plays a major part in lead metabolism, and is one of the most sensitive organs to sub-acute/acute exposure to lead (besides the brain) (Salama et al., 2016; Harari et al., 2018). Some scholars have suggested that lead-induced nephrotoxicity includes oxidative stress, the inflammatory response, and histopathologic changes (Liu et al., 2012a).

Lead-induced oxidative stress occurs when the level of reactive oxygen species (ROS) is out of balance with the level of antioxidants in the kidney (Gautam and Flora, 2010; Jiang et al., 2018). ROS overproduction can increase lipid peroxidation, and then inhibit the activity of antioxidant enzymes, such as superoxide dismutase (SOD), glutathione peroxidase (GSH-Px), and catalase (Navarro-Moreno et al., 2009). Furthermore, overproduction of free radicals induced by lead (e.g., hydroxyl radicals, superoxide anion radicals), which are highly reactive to nephron-membrane lipids and deoxyribonucleic acid (DNA), can lead to stress injuries of glomeruli and nephron tubules (Sudjarwo et al., 2019). Malondialdehyde (MDA) is the main product of lipid peroxidation, which can damage biological macromolecules and be used as a biomarker of lead-induced nephrotoxicity (Qu et al., 2019).

The Kelch-like ECH-associated protein 1/nuclear-factor-E2-related factor 2/antioxidant response element (Keap1/Nrf2/ARE) pathway also plays an important part in lead exposure-based oxidative stress (Cao et al., 2020). Nrf2 is an important transcription factor in the antioxidant system, the expression of which can be regulated negatively by lead. Nrf2 then binds to ARE in the cell nucleus and produces the corresponding antioxidant enzymes, such as hemeoxygenase (HO)-1 and GSH-Px (Li et al., 2019).

Inflammation induced by lead involves the recruitment of innate immune cells, which then produce pro-inflammatory cytokines such as interleukin (IL)-1β, IL-6, and tumor necrosis factor (TNF)-α (Turksoy et al., 2019). Moreover, during this inflammatory response, ROS are produced, which can lead to obvious damage to tissues and cells.

Nuclear factor-kappa B (NF-κB) is a protein complex, which is present in the cytoplasm integrated with inactivated p65 and p50 dimer. Under lead stimulation, the NF-κB pathway can be activated, along with phosphorylation of the I-κB, I-κB kinase (IKK) and nuclear translocation of NF-κB p65 (Jiang et al., 2018). Then, activated NF-κB promotes expression of several cytokine genes, such as IL-1β, IL-6 and TNF-α (Rozenberg et al., 2019). Thus, the NF-κB pathway is tightly involved in the pathologic conditions in the kidney and brain exposed to lead (Liu et al., 2012b; Aladaileh et al., 2020).

Several antioxidant and anti-inflammatory pharmaceutical drugs are available for therapy of lead-induced kidney disease, but have undesirable side effects (Gargouri et al., 2020). In recent years, herbal extracts based on traditional Chinese medicine (TCM) have been reported to provide protection against oxidative stress and inflammation. They have emerged as potential therapeutics to lead-induced nephrotoxicity, and the main bioactive components are flavonoids (Wang et al., 2013; Sudjarwo et al., 2019; Rahmi et al., 2020). Flavonoids are a group of polyphenolic compounds, with extensive pharmacologic properties (e.g., antioxidant, anti-inflammatory, hepatoprotective, anticarcinogenic) (Choy et al., 2019; Wang S. W. et al., 2019). The functional oxo groups and hydroxyl groups of flavonoids play a key part in metal-chelating capacities (Wang Q. et al., 2019). Lead-kaempferol- and lead-quercetin-chelating complexes possess strong antioxidant abilities in ferric reducing power assay and ABTS radical-scavenging systems (Wang et al., 2020).

*Smilax glabra* Roxb. is called “tufuling” in TCM (Cai et al., 2015). The rhizome of *S. glabra* has been used for the treatment of poisoning by heavy metals (e.g., lead, mercury), nephritis and, inflammatory diseases (Xia et al., 2010; Lu et al., 2014; Zhao J. W. et al., 2018). The main compounds in *S. glabra* extract are flavonoids; the content is ~687 mg rutin equivalents/g dry extract. Pure total flavonoids from *S. glabra* Roxb have affects against renal fibrosis and epithelial-mesenchymal transition *in vitro* and *in vivo* (Luo et al., 2019). The n-butyl alcohol fraction of *S. glabra* extract has been shown to inhibit the swelling rate of paws and levels of IL-1β, IL-6 and TNF-α in carrageenan-induced arthritic rats (Bao et al., 2018). Previously, we reported that an *S. glabra* extract could increase the activity of antioxidant enzymes significantly, and decrease lead levels in the blood and kidney of Pb exposed adult rats (Xia et al., 2010). In fact, lead-induced toxicity is age-independent in humans, and the highest risk is in children (Nam et al., 2019). However, only limited information is available on the effects of lead on the kidney development of children.

We investigated the protective effects of *S. glabra* flavonoids extract (SGF) against lead-induced nephrotoxicity in weaning rats and human embryonic kidney (HEK)-293 cells. This is the first report suggesting the antioxidant and anti-inflammatory properties of this quality-controlled extract in sub-acute experimental lead poisoning *in vivo* and *in vitro*.

## Materials and Methods

### Ethical Approval of the Study Protocol

The Animal Care and Use Committee of our university approved the protocol for the animal study. Animals were cared for in accordance with the ethical guidelines of Zhejiang Chinese Medical University (Hangzhou, China). Procedures and interventions conformed to *Guidelines for the Care and Use of Laboratory Animals* (National Institutes of Health (NIH), Bethesda, MD, USA).

### Plant Material and Preparation of SGF

The rhizome of *S. glabra* (batch number: 130101) was purchased from Chinese Herbal Medicines Company (Hangzhou, China). It was identified by an herbalist of Zhejiang Chinese Medical University. Meanwhile, a voucher specimen (number: ZCPS7001) was prepared and deposited at the herbarium of Zhejiang Chinese Medical University. SGF was prepared as we described previously (Xia et al., 2013). Briefly, the air-dried powder was extracted thrice with 60% ethanol at 80°C for 2 h each time, and the ethanol from the extract was removed under a vacuum after filtered. Then, the residue was dissolved in distilled water and fractionated with *n*-butanol. The solvents from the fractionated extracts were removed under a vacuum and the residues lyophilized. The dry powder, as SGF, was used for the current study. We undertook quantitative analyses of the components for quality control of SGF. The content of total flavonoids was 687 mg rutin equivalents/g, and the percentage content of the five main flavonoids (astilbin, neoastilbin, isoastilbin, neoisoastilbin, and engeletin) in the dry powder of SGF was 18.10, 11.04, 5.03, 4.09, and 2.58%, respectively (**Supplementary Figures S1 and S2**, **Supplementary Table S1**).

### Animals and Experimental Protocol

Twenty-four male (300 ± 10 g) and 24 female (220 ± 10 g) Sprague-Dawley rats were obtained from SLAC Laboratory Animals (Shanghai, China). Rats were housed in stainless-steel cages and allowed to acclimatize to the experimental facility for 1 week with a circumambient temperature of 23 ± 1°C, a 12 h dark/light cycle and relative humidity of 55 ± 5%. Rats were allowed to eat standard laboratory food and deionized water. One week later, one male rat and one female rat were placed together in one cage for mating; this was done for 24 cages. The vaginal plug of female rats was checked to ascertain if mating had been successful. After 21 days of milk feeding, the young rats were allowed to wean. We selected male weaning rats for further experimentation.

Forty-eight weaning rats were divided randomly into six groups of eight: control (deionized water); 0.5% lead acetate (LA) solution exposed, Pb + meso-2,3-dimercaptosuccinic acid (DMSA, Sigma–Aldrich, Saint Louis, MO, USA) (as a positive control, 0.5% LA solution + 70 mg/kg DMSA dissolved in deionized water), Pb + SGF (0.5% LA solution + 50, 100 or 200 mg/kg SGF dissolved in deionized water). Animals were given different test substances *via* the oral route for 28 days. Body weight was recorded every week. At the end of this experiment, individual urine samples were collected using metabolism cages. Then, anesthesia was induced using 3% pentobarbital sodium, and blood samples collected from the abdominal aorta. The left kidney was weighed. Then, a portion of it was excised, fixed in 10% formalin for histology examination, and the remainder used for determination of the lead level, cytokines level, as well as biochemical and oxidative parameters.

### Determination of Lead Concentrations and Biochemical Parameters

Kidney samples were weighed and digested with nitric acid at 135°C using a MARS microwave digestion system (CEM, Matthews, NC, USA). The lead concentration in blood, urine and kidney was measured using a quadrupole inductively coupled plasma-mass spectrometer (Aurora M90; Bruker, Billerica, MA, USA).

The level of serum uric acid (UA), serum creatinine (SCr), blood urea nitrogen (BUN), and urinary creatinine (UCr) was measured according to manufacturer instructions (Nanjing Jiancheng Bioengineering Institute, Nanjing, China; or Desai Diagnostic System, Shanghai, China; batch number: 20160308) *via* an automatic biochemical analyzer (Hitachi, Tokyo, Japan). The glomerular filtration rate (GFR) was estimated by calculating creatinine clearance. This was done using 24 h urine volume as well as serum and urine concentrations of creatinine *via* the formula (Shirpoor et al., 2016; Oosterhuis et al., 2017).

$$GFR=[(\text{UCr}\times V_{24\text{h}}/1440)/SCr]$$

### Determination of Parameters of Oxidative Stress and Pro-Inflammatory Cytokines

Kidney tissues were homogenized and dissolved in extraction buffer. The level of ROS, MDA and GSH and the activity of antioxidant enzymes (SOD, CAT and GSH-Px) were measured using test kits from Nanjing Jiancheng Bioengineering Institute (batch number: 20160127) following manufacturer instructions.

The level of the pro-inflammatory cytokines IL-1β, IL-6, and TNF-α in kidney homogenates (10%) was measured using enzyme-linked immunosorbent assay (ELISA) kits following manufacturer instructions (batch number: 155846050; eBioscience, San Diego, CA, USA). The absorbance from each sample was detected at 450 nm.

### Histology of Kidney Tissue

Kidney samples were resected and immersed in 10% formalin. These kidney samples were hydrated in increasing grades of ethanol, cleared in xylene, and embedded in paraffin. Then, sections of thickness 5 μm were sliced and stained with hematoxylin and eosin (H&E) for histology.

### Culture of HEK-293 Cells

HEK-293 cells (American Type Tissue Collection, Manassas, VA, USA) were cultured in Dulbecco’s modified Eagle’s medium (DMEM, Hyclone, Logan, UT, USA) supplemented with 10% fetal bovine serum (Gibco, Carlsbad, CA, USA) at 37°C in an atmosphere containing 5% carbon dioxide. HEK-293 cells were allowed to adapt for 24 h before treatment in all experiments.

### Viability Assay and Morphology Observation of HEK-293 Cells

Cell viability was measured by the Cell Counting Kit (CCK)-8 assay (batch number: 1755C459; Biosharp Life Sciences, Beijing, China) in accordance with manufacturer instructions. HEK-293 cells were treated with LA (0, 25, 50, 100, 200, 400, 800, and 1,600 μM; Sigma–Aldrich) for 3, 12, 24, or 48 h. Eight concentrations of SGF and DMSA (as a positive drug; 0, 25, 50, 100, 200, 400, 800, and 1,600 μg/mL; Sigma–Aldrich) were employed to determine the optimal cytoprotective concentration of SGF and DMSA for 24 h. Moreover, HEK-293 cells were seeded into 96-well plates (2×10^4^ cells/well) for 24 h and treated with SGF (final concentration: 50, 100, and 200 μg/mL) and DMSA (50 μg/mL) for 1 h, then exposed to LA (200 μM) for 24 h. Next, CCK-8 solution was added to each well at 37°C for 1 h. Finally, absorption values were determined at 450 nm using a multi-well plate reader (Biotek, Vinooski, VT, USA).

To analyze morphologic modifications of HEK-293 cells in the presence of Pb with or without SGF, cells were observed under an inverted microscope (Olympus, Tokyo, Japan) after 24 h of treatment.

### Apoptosis of HEK-293 Cells Using Fluorescence Microscopy and Flow Cytometry

Damage to the DNA of HEK-293 cells was analyzed by the terminal deoxynucleotidyl transferase dUTP nick-end labeling (TUNEL) assay. Cells were stimulated with lead for 24 h after pretreatment with SGF and DMSA for 1 h. Then, they were fixed in 4% paraformaldehyde in phosphate-buffered saline (PBS) and processed for TUNEL staining using the One Step TUNEL Apoptosis Assay Kit (Yeasen Biotechnology, Shanghai, China) as described previously (Li et al., 2020). Proteinase K was used to permeabilize cells according to manufacturer instructions. DNA staining was undertaken with 4′,6-diamidino-2-phenylindole (DAPI). TUNEL signals were observed with a fluorescence microscopy using 488 nm and 520 nm (Nikon, Tokyo, Japan). The percentage of TUNEL-positive cells was determined as the ratio of the number of TUNEL-positive cells in at least three random fields to that of total cells, followed by multiplication by 200.

We also measured the rate of apoptosis of HEK-293 cells apoptosis by flow cytometry using the Annexin V-FITC/PI Apoptosis Kit (batch number: C1063; Beyotime Institute of Biotechnology, Shanghai, China). Cells were stimulated with Pb for 24 h after pretreatment with SGF and DMSA for 1 h. Then, cells were collected and double-stained with Annexin V-fluorescein isothiocyanate/propidium iodide (FITC/PI) and analyzed by a flow cytometer (BD Biosciences, Franklin Lakes, NJ, USA). For further studies, the level of the key proteins involved in kidney-cell apoptosis (caspase-3 and cleaved caspase-3) was measured by western blotting. Antibodies were supplied from Cell Signaling Technology (Boston, MA, USA) and the catalog number was 9662S and 9661S, respectively.

### Measurement of Levels of ROS and Antioxidant Parameters

HEK-293 cells were seeded into 96-well plates (2×10^4^ cells/well). Cells were stimulated with LA (200 μM) for 24 h after pretreatment with SGF and DMSA for 1 h. 2′,7′-dichlorofluorescein diacetate (DCFH-DA) (10 μM; batch number: 20180804; Nanjing Jiancheng Bioengineering Institute) was added and incubation allowed for an additional 30 min at 37°C in the dark. Next, cells were washed twice with PBS and resuspended in PBS, followed by analyzed by fluorescence microscope.

HEK-293 cells were homogenized and dissolved in extraction buffer to analyze the antioxidant parameters. The level of MDA and GSH and the activity of the antioxidant enzymes SOD, CAT and GSH-Px in HEK-293 cells were determined using test kits (batch number: 20180804) from Nanjing Jiancheng Bioengineering Institute according to manufacturer instructions.

### Assay to Measure the Level of Pro-Inflammatory Cytokines

The method used to measure the level of the pro-inflammatory cytokines IL-1β, IL-6 and TNF-α in the supernatants of HEK-293 cells was identical to that employed for kidney cells (batch number: 158611029). Briefly, HEK-293 cells were grown in six-well plates (10^6^ cells/well) for 24 h, and then treated with SGF and DMSA for 1 h before exposure to LA (200 μM). After stimulation with LA for 24 h, cell-free supernatants were collected for analysis of secretion of IL-1β, IL-6 and TNF-α.

### Western Blotting for Protein Expression of the Keap1/Nrf2/HO-1 Pathway and NF-κB Pathway

Total proteins were extracted using standard methods. The protein concentration was measured using a Bicinchoninic Acid Protein Assay Kit (batch number: 00171509; Kangwei Century Biotechnology, Beijing, China). Subsequently, 40 µg of protein was loaded onto 8 or 12% gels for sodium dodecyl sulfate–polyacrylamide gel electrophoresis. Proteins were transferred onto polyvinylidene fluoride (PVDF) membranes. After transfer, membranes were blocked with 5% bovine serum albumin (Sigma–Aldrich) in 1× TBST (Tris-buffered saline and Tween-20) buffer at room temperature for 2 h. PVDF membranes were incubated with primer antibody at 4°C overnight, and then secondary antibody at room temperature for 2 h according to manufacturer instructions. Fluorescence signals were measured and analyzed using Odyssey Imager (LI-COR Biosciences, Lincoln, NB, USA). ImageJ 1.41 (NIH) was used for calculation of absorbance.

The main antibodies used in the experiments were supplied from Cell Signaling Technology: Keap1 (batch number: 8047S1901), Nrf2 (12721S1903), HO-1 (70081S1901), NF-κB p65 (8242S1902), phospho-NF-κB p65 (3033S1901), IκBα (4812S1902), phospho-IκBα (2859S1901), IKKα (2682S1902), phospho-IKKα (2697S1902), and β-actin (3700S1901).

To investigate the effect of SGF on activation of NF-κB p65, the proteins of nuclear fraction and cytoplasmic fraction were extracted with the NE-PER™ Kit (Thermo Scientific, Waltham, MA, USA). Western blotting was conducted as mentioned above, and PVDF membranes were incubated with primary antibodies. Histone H3 (batch number: 4499S2004; Cell Signaling Technology was the reference protein for the cell nucleus. The rinse and fluorescence signals analysis of membranes were performed as mentioned above.

### Effects of SGF on Nuclear Translocation of NF-κB p65 Protein

HEK-293 cells were immunofluorescence-labeled according to manufacturer instructions using a Cellular NF-κB Translocation Kit (aliquot batch number: 16; Beyotime Institute of Biotechnology, Shanghai, China). Briefly, HEK-293 cells were cultivated with a blocking solution for 1 h to restrain non-specific binding after washing and fixing. Then, cells were incubated with NF-κB p65 primary antibody at 4°C overnight. Next, HEK-293 cells were cultivated with a Cy3-conjugated secondary antibody for 1 h. Finally, cells were cultivated with DAPI for 5 min. Pink ﬂuorescence (red and blue images overlaid) indicated nuclear translocation of NF-κB p65 protein, and could be observed simultaneously by a confocal laser scanning microscope (Zeiss, Wetzlar, Germany) at an excitation wavelength of 350 nm and 540 nm for DAPI and Cy3 (Oh et al., 2019).

### Statistical Analyses

Results were analyzed by one-way analysis of variance, followed by a *post hoc* multiple-comparison test using the SPSS 20.0 (IBM, Armonk, NY, USA). Data are the mean ± SD. *P* < 0.05 was considered significant.

## Results

### Effects of SGF on Body Weight, Kidney/Body Ratio, and Lead Concentration of Weaning Rats With Lead-Induced Nephrotoxicity

At the end of animal experiment, Pb exposed rats lost significantly more weight compared with that of rats in the control group (**Table 1**). However, SGF administration resulted in significant increase in the body weight and decreased kidney/body ratio compared with that in the Pb exposed group. SGF and DMSA were effective in reducing the lead concentration in blood and kidney tissue compared with that in Pb exposed rats. Moreover, SGF had an obvious effect on facilitating urinary lead excretion in a dose-dependent manner.

**Table 1:** Effects of SGF on body weight, kidney/body ratio, lead concentration in blood, urine and kidney of rats with lead-induced nephrotoxicity.

Group	Body weight increase (g)	Kidney/body ratio (mg/g)	Blood lead(μg/100 mL)	Urine lead(μg/100 mL)	Kidney lead(μg/100 g)
Control	201 ± 18.2^a^	7.1 ± 0.82^c^	0.18 ± 0.07^e^	7.97 ± 0. 81^d^	0.41 ± 0.01^e^
Pb exposed	176 ± 16.9^b^	11.3 ± 1.03^a^	10.21 ± 0.93^a^	250.47 ± 19.48^a^	32.09 ± 0.31^a^
Pb + DMSA	202 ± 19.7^a^	8.6 ± 0.91^c^	0.83 ± 0.14^d^	129.36 ± 22.10^b^	4.2 ± 0.07^d^
Pb + SGF-50	194 ± 18.8^a^	10.0 ± 0.98^b^	5.45 ± 0.40^b^	83.86 ± 9.07^c^	18.5 ± 0.15^b^
Pb + SGF-100	198 ± 20.1^a^	9.4 ± 0.74^b^	4.95 ± 0.34^b^	97.20 ± 7.29^c^	16.2 ± 0.06^b^
Pb + SGF-200	200 ± 19.4^a^	8.9 ± 0.85^c^	3.83 ± 0.23^c^	107.64 ± 10.55^b^	13.6 ± 0.05^c^

The results are expressed as the mean ± SD, n = 8; Different letters in the same column indicate significant differences (P < 0.05). Pb exposed, 0.5% lead acetate in drinking water; DMSA, meso-2,3-dimercaptosuccinic acid, 70 mg/kg; SGF-50, SGF-100, SGF-200, Smilax glabra flavonoids extract, 50 mg/kg, 100 mg/kg, and 200 mg/kg.

### Effects of SGF on Parameters of Renal Biochemistry and Oxidative Stress of Weaning Rats

**Figure 1:**
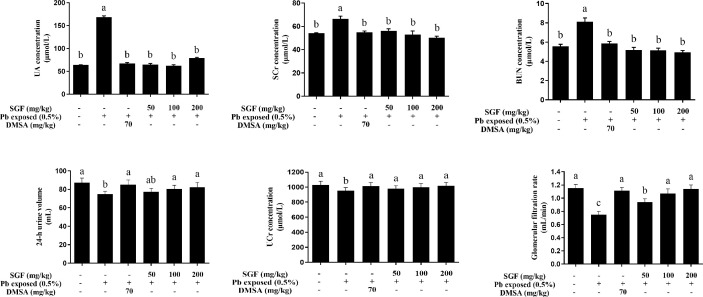
Effects of SGF on renal biochemical parameters of rats with lead-induced nephrotoxicity. The results are expressed as the mean ± SD, n = 8; Different letters indicate significant differences (P < 0.05). UA, uric acid; BUN, urea nitrogen; SCr, serum creatinine; UCr, urine creatinine; GFR, glomerular filtration rate; DMSA, meso-2,3-dimercaptosuccinic acid; SGF, *Smilax glabra* flavonoids extract.

UA, SCr, BUN, and UCr were used as biochemical markers for weaning rats with lead-induced nephrotoxicity. There was a significant increase in the level of UA, SCr and BUN in the Pb exposed group compared with that in the control group, indicating that lead induced significant damage to the kidney. SGF (50, 100, and 200 mg/kg) reduced the level of UA, SCr, and BUN markedly in a dose-dependent manner compared with that in the Pb exposed group. In the Pb exposed group, the GFR was significantly lower than that in the control group. SGF treatment increased the GFR significantly compared with that in the Pb exposed group, but the GFR in the SGF group of 50 mg/kg remained significantly lower than that in the control group (**Figure 1**).

**Figure 2:**
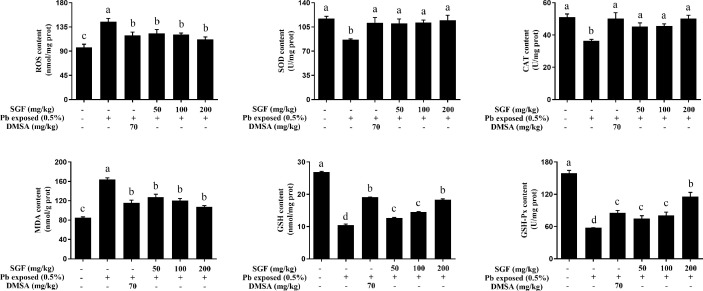
Effects of SGF on renal ROS level and oxidative stress parameters of rats with lead-induced nephrotoxicity. The results are expressed as the mean ± SD, n = 8; Different letters indicate significant differences (P < 0.05). ROS, reactive oxygen species; SOD, superoxide dismutase; CAT, catalase; MDA, malondialdehyde; GSH, glutathione; GSH-Px, glutathione peroxidase; DMSA, meso-2,3-dimercaptosuccinic acid; SGF, *Smilax glabra* flavonoids extract.

Oxidative stress played a key part in lead-induced renal damage in weaning rats. Hence, we examined if SGF administration could inhibit ROS production and lead-triggered oxidative stress. Compared with the Pb exposed group, SGF could significantly increase the activity of SOD, CAT and GSH-Px and the GSH level, and obviously decrease ROS generation and the MDA level in the kidney tissues of weaning rats with lead-induced nephrotoxicity (**Figure 2**). DMSA also showed obvious renal-protection and antioxidant capacities.

### Effects of SGF on the Level of Pro-Inflammatory Cytokines of Weaning Rats

SGF administration significantly reduced lead-triggered increase in the level of IL-1β, IL-6, and TNF-α in the kidney of weaning rats with lead-induced nephrotoxicity (**Table 2**). In fact, the anti-inflammatory activity of SGF had a dose-dependent effect on these pro-inflammatory cytokines. DMSA was also a good anti-inflammatory agent.

**Table 2:** Effects of SGF on pro-inflammatory cytokines levels in the kidney tissue of rats with lead-induced nephrotoxicity.

Group	IL-1β (ng/mL)	IL-6 (ng/mL)	TNF-α (ng/mL)
Control	0.05 ± 0.00^f^	0.14 ± 0.01^f^	0.12 ± 0.01^f^
Pb exposed	0.42 ± 0.04^a^	1.39 ± 0.12^a^	1.05 ± 0.09^a^
Pb + DMSA	0.12 ± 0.01^e^	0.31 ± 0.03^e^	0.23 ± 0.02^e^
Pb + SGF-50	0.29 ± 0.02^b^	0.84 ± 0.07^b^	0.68 ± 0.07^b^
Pb + SGF-100	0.23 ± 0.02^c^	0.65 ± 0.05^c^	0.50 ± 0.04^c^
Pb + SGF-200	0.17 ± 0.01^d^	0.52 ± 0.06^d^	0.38 ± 0.04^d^

The results are expressed as the mean ± SD, n = 8; Different letters in the same column indicate significant differences (P < 0.05). Pb exposed, 0.5% lead acetate in drinking water; DMSA, meso-2,3-dimercaptosuccinic acid, 70 mg/kg; SGF-50, SGF-100, SGF-200, Smilax glabra flavonoids extract, 50 mg/kg, 100 mg/kg, and 200 mg/kg.

### Effects of SGF on Renal Histologic Changes in Weaning Rats

**Figure 3:**
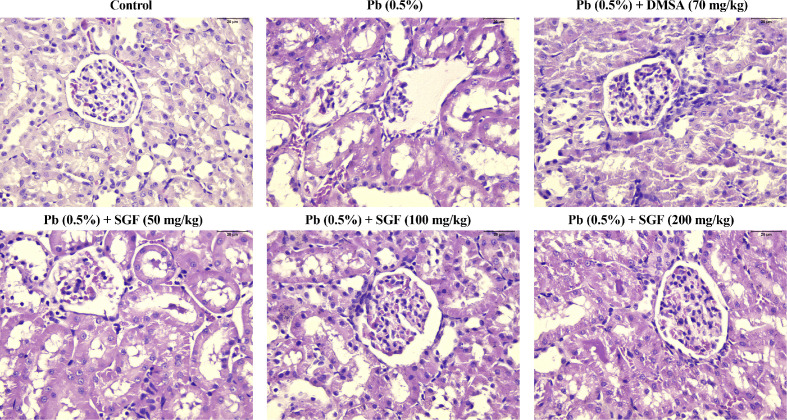
Effects of SGF on histological change of kidney in rats with lead-induced nephrotoxicity (400× H&E stain). DMSA, meso-2,3-dimercaptosuccinic acid; SGF, *Smilax glabra* flavonoids extract.

Kidney tissue showed no significant histopathologic changes in rats of the control group (**Figure 3**). The kidney of rats treated with Pb alone showed tubular dilatation, vacuolar, hemorrhage, glomerulus hypercellularity and cellular debris. SGF administration led to a significant improvement in renal pathologic alterations induced by Pb. In particular, SGF (200 mg/kg) could significantly improve the pathological changes in the renal tissue of rats with lead-induced nephrotoxicity. Rats treated with DMSA showed, in general, normal renal tubules and glomerulus.

### Effects of SGF on Cytotoxicity of Pb Exposed HEK-293 Cells

**Figure 4:**
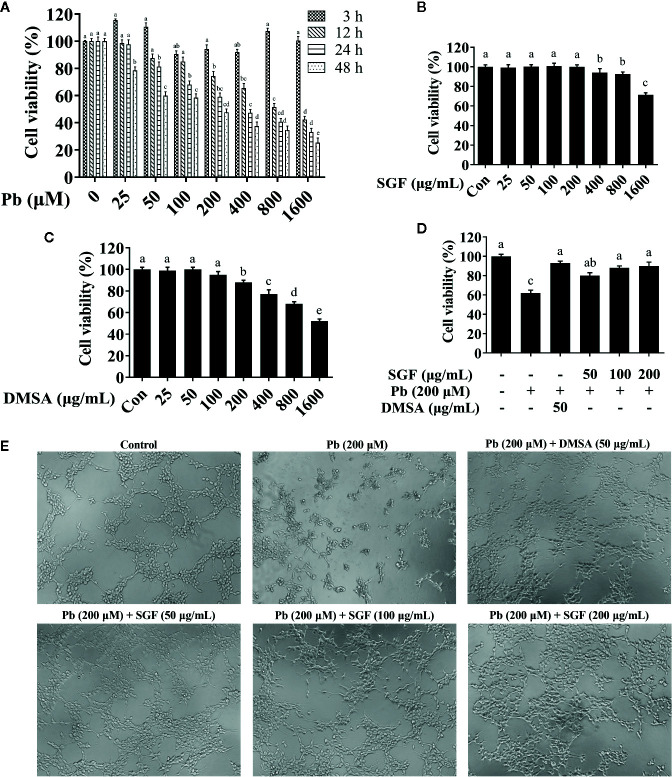
Effects of SGF and DMSA on HEK-293 cells viability. The results are expressed as the mean ± SD, n = 3; Different letters indicate significant differences (P < 0.05). **(A)** Lead cytotoxicity to HEK-293 cells; **(B, C)** Effects of SGF and DMSA on cell viability in HEK-293 cells for 24 h; **(D)** Cell viability measured by a CCK-8 assay; **(E)** HEK-293 cells were stimulated with lead for 24 h after pretreated with SGF and DMSA for 1 h, and cells were observed with inverted microscope (100×). DMSA, meso-2,3-dimercaptosuccinic acid; SGF, *Smilax glabra* flavonoids extract.

To choose an appropriate concentration for further study in cell tests, the effects of different doses of Pb, SGF and DMSA on cell viability were assessed using the CCK-8 assay (**Figure 4**). We found that LA at 200 µM had a significant toxic effect upon HEK-293 cells for 24 h, and this concentration was selected for the further study (**Figure 4A**). We chose 50, 100, or 200 μg/mL as the concentrations of SGF for subsequent experiments because they were not toxic to HEK-293 cells (**Figure 4B**). The appropriate DMSA concentration (50 μg/mL) was chosen for subsequent experiments because it was the dose that did not induce an overt detrimental effect on cell viability (**Figure 4C**). Subsequently, SGF doses of 50, 100, or 200 μg/mL and 50 μg/mL DMSA were chosen for pretreatment of HEK-293 cells for 1 h before treatment with LA (200 μM) for another 24 h. To measure the effects of SGF on the viability of lead-treated renal cells, HEK-293 cells were treated with LA (200 µM), LA (200 µM) + DMSA (50 μg/mL), and LA (200 µM) + SGF (50, 100, or 200 μg/mL). We discovered that SGF treatment improved the viability of Pb exposed HEK-293 cells significantly (**Figure 4D**). Inverted microscope revealed the morphologic changes of HEK-293 cells incubated with Pb during 24 h (**Figure 4E**). The number of non-adherent cells increased gradually during Pb exposure. However, HEK-293 cells treated with both Pb and a high concentration of SGF (200 μg/mL) exhibited a morphology close to that of cells in the control group.

### Effects of SGF on the Apoptosis of Pb Exposed HEK-293 Cells

**Figure 5:**
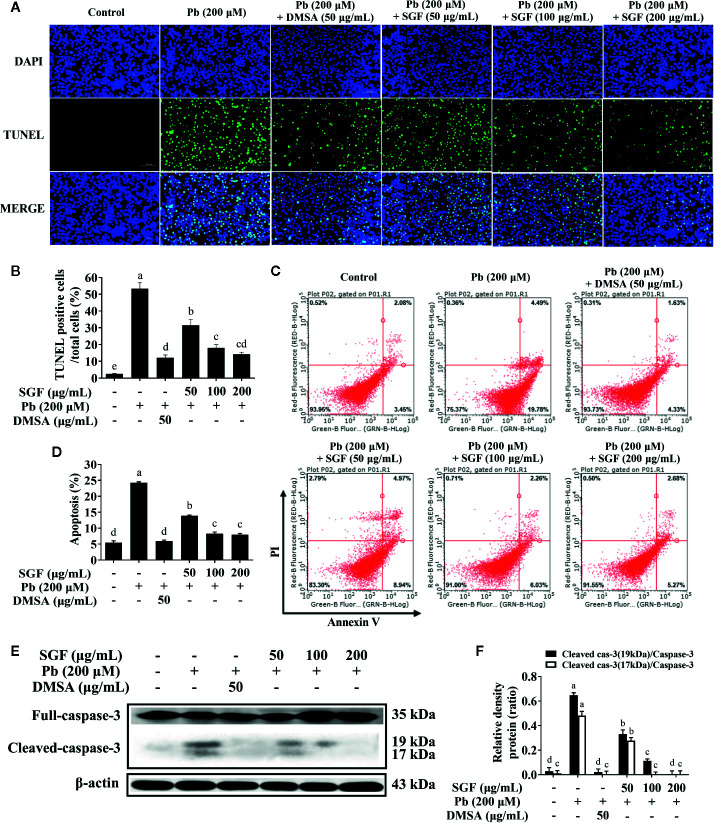
Protective effects of SGF on Pb exposed HEK-293 cells apoptosis. The results are expressed as the mean ± SD, n = 3; Different letters indicate significant differences (P < 0.05). **(A, B)** TUNEL assay (200×); **(C, D)** Flow cytometry analysis; **(E, F)** Western blotting detection of the full- and cleaved-caspase-3 protein expression. DMSA, meso-2,3-dimercaptosuccinic acid; SGF, *Smilax glabra* flavonoids extract.

To further elucidate the effects of SGF on Pb exposed HEK-293 cells, the TUNEL assay was carried out. The TUNEL assay is based on the detection and linking of DNA nick ends by TdT with fluorochrome-labeled dUTP nucleotides. TUNEL-positive cells were observed by fluorescence microscopy (**Figures 5A, B**). Compared with the control group, HEK-293 cells treated with Pb alone showed a significant increase in the number of TUNEL-positive cells. Moreover, compared with the model group, lead-induced apoptosis was decreased markedly by SGF treatment according to the TUNEL assay. We used flow cytometry to further study the ability of SGF to reduce the apoptosis of HEK-293 cells. SGF could reduce Pb exposed apoptosis of HEK-293 cells significantly; the amount of Annexin V-FITC/PI-stained cells were reduced significantly upon SGF (50, 100, and 200 μg/mL) treatments in a dose-dependent fashion (**Figures 5C, D**). Lead-induced apoptosis was also verified by caspase-3 and cleaved caspase-3 protein expression. Compared with the control group, expression of cleaved caspase-3 at the protein level was increased in Pb exposed HEK-293 cells. SGF treatment could decrease expression of cleaved caspase-3 significantly, thereby reducing lead-induced apoptosis of HEK-293 cells (**Figures 5E, F** **)**.

### Effects of SGF on Oxidative-Stress Parameters and the ROS-Mediated Keap1/Nrf2/ARE Pathway in Pb Exposed HEK-293 Cells

**Figure 6:**
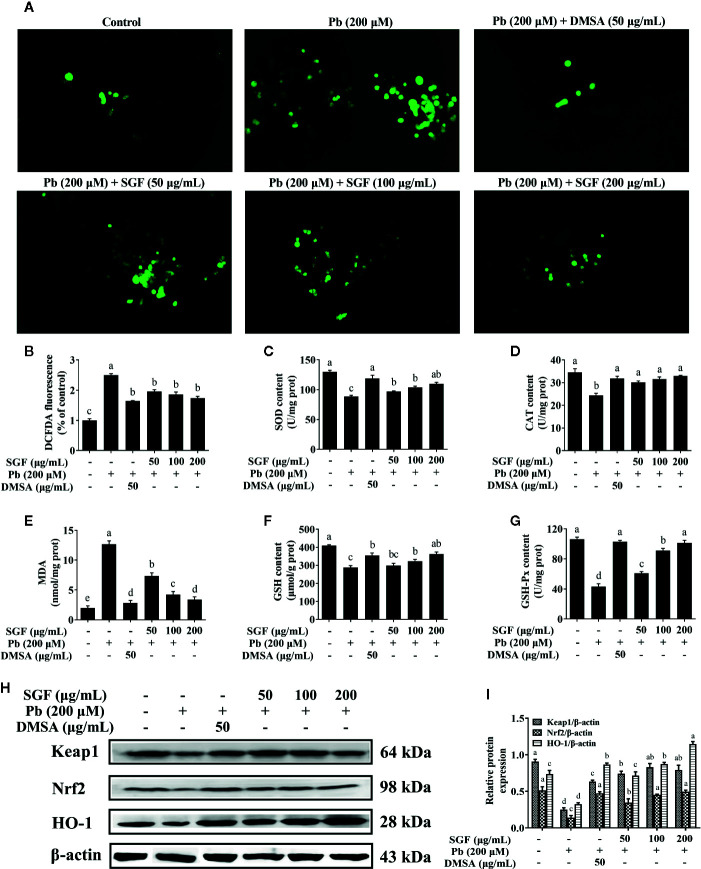
Effects of SGF on ROS accumulation, oxidative stress parameters and Keap1/Nrf2/ARE pathwa in Pb exposed HEK-293 cells. The results are expressed as the mean ± SD, n = 3; Different letters indicate significant differences (P < 0.05). **(A, B)** ROS accumulation detected by fluorescence microscope (100×); **(C–G)** Oxidative stress parameters. SOD, superoxide dismutase; CAT, catalase; MDA, malondialdehyde; GSH, glutathione; GSH-Px, glutathione peroxidase; Values were expressed as mean ± SD, n = 10; (**H, I**) Western blotting detection of the Keap1, Nrf2 and HO-1 protein expression; Values were expressed as mean ± SD, n = 3. DMSA, meso-2,3-dimercaptosuccinic acid; SGF, *Smilax glabra* flavonoids extract.

The fluorescence intensity of DCFDA was measured by fluorescence microscope (**Figures 6A, B**). Pb exposed HEK-293 cells showed a significant increase in ROS level compared with that in the control group. Treatment with SGF (50, 100, and 200 μg/mL) decreased the ROS level significantly in a dose-dependent manner compared with that in Pb exposed HEK-293 cells. The activities of antioxidant enzymes can be seen as an index of the antioxidant status of the kidney (Shenai-Tirodkar et al., 2017). We showed that Pb induced a marked depletion in the antioxidant capacity as reflected by the reduced activity of SOD (**Figure 6C**), CAT (**Figure 6D**), GSH-Px (**Figure 6G**) and reduced level of GSH (**Figure 6F**) compared with that in control cells. The oxidative-stress parameter, MDA content, was increased significantly in Pb exposed HEK-293 cells (**Figure 6E**). Conversely, compared with the Pb exposed group, SGF could notably increase the GSH level and activity of SOD, CAT, and GSH-Px, and obviously decrease MDA level of Pb exposed HEK-293 cells. To further explore the mechanisms of SGF reduction of ROS accumulation in HEK-293 cells treated with Pb, we detected the effect of SGF on Nrf2 (a key regulator of redox homeostasis in cells). It has been reported that the Keap1/Nrf2/ARE signaling pathway plays an important part in ROS-induced oxidative stress (Yamawaki et al., 2018; Upadhyaya et al., 2019). We measured expression of the proteins related to this pathway by western blotting. Compared with the control group, expression of Keap1, Nrf2 and HO-1 at the protein level was reduced in Pb exposed HEK-293 cells. Nevertheless, SGF treatment could increase expression of Keap1, Nrf2, HO-1 significantly, thereby reducing oxidative injury to HEK-293 cells (**Figures 6H, I**).

### Effects of SGF on Levels of Pro-Inflammatory Cytokines and the NF-κB Pathway in Pb Exposed HEK-293 Cells

**Figure 7:**
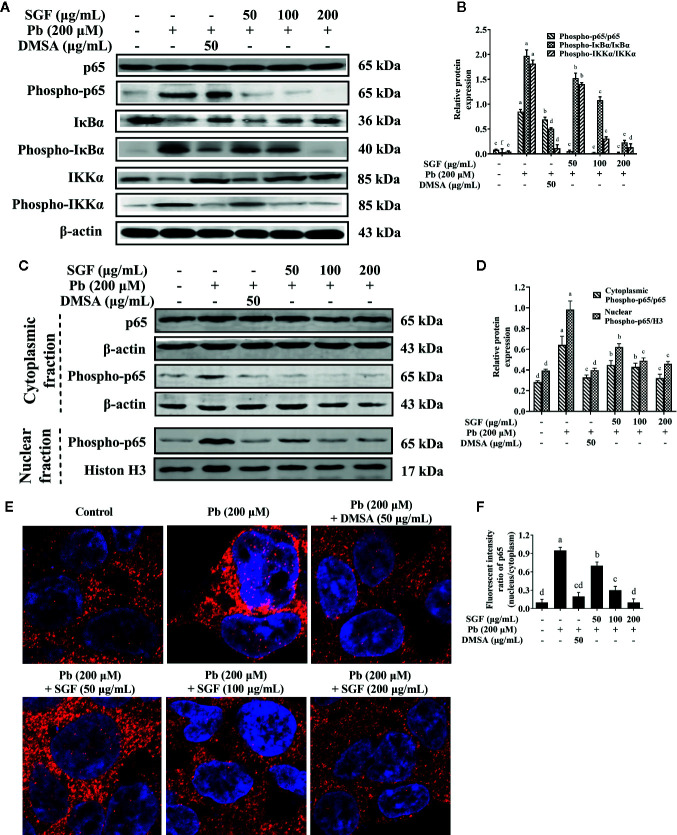
Effects of SGF on pro-inflammatory NF-κB pathway and p65 protein nuclear translocation in Pb exposed HEK-293 cells. The results are expressed as the mean ± SD, n = 3; Different letters indicate significant differences (P < 0.05). **(A, B)** Western blotting detection of the p65, phospho-p65, IκBα, phospho-IκBα, IKKα and phospho-IKKα expression; **(C, D)** Western blotting detection of phospho-p65 expression in nucleus and p65, phospho-p65 expression in cytoplasm. **(E, F)** Determination of NF-κB p65 protein nuclear translocation by laser confocal microscope (1,260×). DMSA, meso-2,3-dimercaptosuccinic acid; SGF, *Smilax glabra* flavonoids extract.

SGF reduced the level of IL-1β, IL-6 and TNF-α significantly compared with that of Pb exposed HEK-293 cells (**Table 3**). Protein expression of phospho-p65, phospho-IκBα and phospho-IKKα increased significantly in Pb exposed HEK-293 cells compared with that in control cells (**Figures 7A, B**). Moreover, nuclear expression of phospho-p65 was up-regulated significantly in the Pb exposed group compared with that in the control group (**Figures 7C, D**). SGF could obviously decrease expression of these proteins. In particular, higher concentrations of SGF (100 and 200 μg/mL) seemed to have greater effects than those of a lower dose (50 μg/mL) involved in the NF-κB/IκB/IKK pathway.

**Table 3:** Effects of SGF on pro-inflammatory cytokines levels in Pb exposed HEK-293 cells.

Group	IL-1β (ng/mL)	IL-6 (ng/mL)	TNF-α (ng/mL)
Control	0.02 ± 0.00^f^	0.03 ± 0.00^f^	0.10 ± 0.01^e^
Pb exposed	0.12 ± 0.01^a^	0.16 ± 0.02^a^	0.65 ± 0.07^a^
Pb + DMSA	0.04 ± 0.00^e^	0.05 ± 0.00^e^	0.18 ± 0.02^d^
Pb + SGF-50	0.07 ± 0.01^b^	0.11 ± 0.01^b^	0.39 ± 0.04^b^
Pb + SGF-100	0.06 ± 0.00^c^	0.09 ± 0.01^c^	0.31 ± 0.03^b^
Pb + SGF-200	0.05 ± 0.00^d^	0.07 ± 0.00^d^	0.24 ± 0.03^d^

The results are expressed as the mean ± SD, n = 3; Different letters in the same column indicate significant differences (P < 0.05). Pb exposed, lead acetate, 200 µM; DMSA, meso-2,3-dimercaptosuccinic acid, 50 µg/mL; SGF-50, SGF-100, SGF-200, Smilax glabra flavonoids extract, 50 µg/mL, 100 µg/mL, and 200 µg/mL.

SGF could reduce lead-induced nuclear translocation of NF-κB p65 according to confocal laser scanning microscope. Pink ﬂuorescence indicated the location of the p65 protein in nuclei (**Figures 7E, F**). The intensity of pink ﬂuorescence in lead-induced HEK-293 cells was significantly higher than that in other groups. These results showed that Pb induced a significant increase in the level of nuclear translocation of NF-κB p65 in HEK-293 cells compared with that in control cells. Treatment with SGF (50, 100, and 200 μg/mL) significantly decreased the nuclear translocation of NF-κB p65 compared with that in the Pb exposed group. These findings confirmed the regulatory effects of SGF on the NF-κB pathway.

## Discussion

Lead is a widespread environmental pollutant that is known to induce a wide range of biochemical and physiological dysfunctions both in humans and in laboratory animals. Children are more sensitive to lead toxicity than adults, which is why we chose young rats instead of adult rats for experimentation.

In recent years, studies have shown that traditional Chinese medicine (TCM) and medicinal plants can remove lead ion from blood and body organs (Xia et al., 2010; Aladaileh et al., 2020; Wang et al., 2020). *S. glabra* as a TCM, has been used for the treatment of lead poisoning over hundreds of years ago in China. The present study showed that *S. glabra* flavonoids extract (SGF) had a certain antagonism to lead in the blood and kidneys of weaning rats with lead-induced nephrotoxicity (**Table 1**). This result is in accordance with data from a study by Adhikari and colleagues. They discovered that a soluble Pb-flavonoids (naringin) complex could reduce lead toxicity *in vivo* and *in vitro* due to the chelation and antioxidant activity of naringin (Adhikari et al., 2018). In the human body, the highest level of lead is found in the kidneys, then the liver, and other soft tissues (Ravipati et al., 2019). The detoxification of xenobiotics by the kidney is mainly through urinary excretion (Gargouri et al., 2019). We also found that SGF has an obvious effect on facilitating urinary lead excretion in a dose-dependent manner. In clinical medicine, the primary goal of treatment for lead-induced toxicity is to promote its excretion using chelating agents (Nam et al., 2019). Our results showed that the flavonoids in *S. glabra* had the potential as effective metal chelators. Excessive accumulation of lead can decrease body growth and increase organs (e.g., kidney, liver) weight (Hou et al., 2019). We showed that SGF could increase the body weight and reduce the kidney weight of Pb exposed rats, which were near to those of the control group.

Lead-induced nephrotoxicity is getting more and more attention in developing countries. UA, SCr and BUN are renal-function indicators. In most instances, abnormal increases in the level of UA, SCr and BUN are caused by kidney damage (Sudjarwo et al., 2019). The present study showed that the levels of UA, SCr and BUN in rats exposed to lead are significantly increase compared with the control group. SGF could decrease the contents of these renal-function indicators significantly (**Figure 1**). Moreover, renal function is represented mainly by glomerular filtration (GF), which is dependent upon the number and function of the nephrons (Shirpoor et al., 2016). Lead aggregated in renal tissue could cause direct damage to glomerular structures and reduce the GFR (**Figure 1**). SGF could protect glomerular structures by increasing the GFR, this result is in accordance with the results of a clinical investigation indicating that the flavonoids quercetin had an obvious glomerular structure-protective effect in patients with iodinated contrast-induced nephropathy, as evidenced by a decreasing level of SCr and increasing GFR (Vicente-Vicente et al., 2019). Moreover, the kidney of weaning rats treated with Pb alone showed obvious damage, SGF could lead to a significant improvement in renal pathologic alterations induced by Pb (**Figure 3**). These results indicated that SGF could be an effective renal-protection agent.

In addition to renal-function indicators and histological changes, oxidative stress and the inflammatory response are believed to be the main mechanisms underlying the nephrotoxicity of Pb exposure (Liu et al., 2012a). Lead participate in a Fenton-like reaction to produce ROS, which induce oxidative stress (Qu et al., 2019). Thus, lead-induced renal oxidative stress is mediated by ROS formation (Gautam and Flora, 2010; Jiang et al., 2018). The antioxidant systems in the body can scavenge ROS (e.g., superoxide radicals, hydroxyl radicals, peroxide radicals, nitric oxide)-induced harmful effects, and reset the oxidant-antioxidant equilibrium in blood and organs (Pastaci et al., 2018). An increase in oxidative stress also causes cell death and oxidation of macromolecule (Sas et al., 2018; Bonaterra et al., 2020). SOD, CAT, GSH-Px are important antioxidant enzymes; they constitute a mutually supportive defense mechanism against ROS (Sudjarwo et al., 2019). We showed that lead acetate could induce obvious oxidative stress in the kidneys of weaning rats (**Figure 2**). SGF treatment induced a dramatic increase in GSH content and activity of SOD, CAT and GSH-Px, and caused a decrease in the level of MDA and ROS in the kidneys of weaning rats.

Lead can also up-regulate expression of pro-inflammatory cytokines, and then cause inflammation (Gupta et al., 2010). Although the pro-inflammatory effect of lead has been observed in many cells, tissues and organs, few studies have focused on the kidney. We demonstrated that lead-induced an increase in expression of IL-1β, IL-6 and TNF-α in the kidneys of weaning rats, and that SGF treatment reduced secretion of IL-1β, IL-6 and TNF-α *in vivo* (**Table 2**).

In the animal experiments mentioned above, we measured the lead concentrations in blood, urine and kidney, the biochemical parameters, the histological changes, the oxidative stress parameters, and the pro-inflammatory cytokines in the kidneys of weaning rats. After confirmed the renal protective effects of SGF *in vivo* model, we further sought to investigate the underlying mechanisms based on the cell model. We aimed to select a stable human cell line for multiple passages so that to perform more in-depth and comprehensive research, such as gene knockout. Thus, prior to the main study, we performed a pilot study to screen the cell among HK-2, HEK-293 and HKC cell lines. After comprehensive comparison of these three cell lines to lead and SGF sensitivities, we chose HEK-293 cell finally. Before evaluating the protective effects of SGF on Pb exposed HEK-293 cells, we assessed the influence of SGF on the viability and morphology of cells, and then selected the Pb exposure concentration and safety dose of SGF and DMSA. We chose lead acetate at 200 µM and SGF at 50, 100 or 200 μg/mL as the concentrations for subsequent experiments in HEK-293 cells (**Figure 4**).

Previous studies indicated that apoptosis is associated with lead-induced toxicity in neuronal cells and leukemia cells (Yedjou et al., 2015). Here, we aimed to assay the lead-induced apoptosis in kidney cells, and evaluate the protection effects of SGF. TUNEL-positive cells were observed by fluorescence microscope (**Figure 5A**). The TUNEL assay demonstrated apoptosis induction. Compared with the control group, HEK-293 cells treated with lead alone showed a significant increase in the number of TUNEL-positive cells. Moreover, compared with the model group, lead-induced apoptosis was decreased markedly by SGF treatment according to the TUNEL assay. Furthermore, we used flow cytometry to study the ability of SGF to reduce apoptosis of HEK-293 cells. SGF could reduce Pb exposed apoptosis of HEK-293 cells significantly, and the number of Annexin V-FITC/PI-stained cells was reduced significantly upon SGF (50, 100 and 200 μg/mL) treatment in a dose-dependent manner (**Figures 5C, D**). This hypothesis was verified by protein expression of caspase-3 and cleaved caspase-3 protein (**Figures 5E, F**). The increased expression of cleaved caspase-3 represents activation of effector caspases, indicating apoptosis (Zhao H. et al., 2018). We showed that Pb exposure caused obvious apoptosis of HEK-293 cells. However, SGF could decrease the apoptosis of Pb exposed HEK-293 cells significantly at test dose due (at least in part) to inhibition of cleavage of pro-caspase-3 into cleaved caspase-3 (**Figure 5E**). The results confirmed that SGF could protect against lead-induced nephrotoxicity by reducing apoptosis.

In order to continue exploring the mechanisms of antioxidant and anti-inflammatory effects of SGF, the proteins expression of related signaling pathway in HEK-293 cells were measured. Certainly, the oxidative stress parameters and pro-inflammatory cytokines were also determined in HEK-293 cells like the animal experiment. Our results showed that lead could induce obvious oxidative stress in HEK-293 cells (**Figure 6**). SGF treatment induced a dramatic increase in GSH content and activity of SOD, CAT, and GSH-Px, and caused a decrease in the level of MDA and ROS in HEK-293 cells. The Keap1/Nrf2/ARE pathway mediates transcription of several antioxidant genes, and natural flavonoids have been reported to increase expression of the genes and proteins related to this pathway (Xiao et al., 2018). The expression of Keap1, Nrf2, and ARE can be regulated negatively by lead. In this study, we showed that SGF is an activator of the Keap1/Nrf2/ARE pathway, and could strongly increase the expression of Keap1, Nrf2, and HO-1 in Pb exposed HEK-293 cells (**Figures 6H, I**). Hence, enhancement of Nrf2 activation may contribute to the antioxidant effect of SGF on kidney cells.

We also demonstrated that lead-induced an increase in expression of IL-1β, IL-6 and TNF-α in HEK-293 cells, and that SGF treatment reduced secretion of IL-1β, IL-6 and TNF-α *in vitro* (**Table 3**). The NF-κB pathway plays a vital part in the inflammatory response, thereby making it an important drug target for treatment of inflammatory diseases (Nakano et al., 2006). Expression of NF-κB p65 has been shown to be increased prominently in lead-induced hypertensive rats, thereby showing that lead can stimulate activation of the NF-κB pathway (Bravo et al., 2007). In fact, the NF-κB pathway can be activated by pro-inflammatory factors such as IL-1β, IL-6 and TNF-α (Gupta et al., 2010). Western blotting showed that phosphorylation of the proteins of NF-κB p65, IκBα and IKKα increased significantly in Pb exposed HEK-293 cells (**Figures 7A, B**), a finding that is in accordance with the excessive secretion of pro-inflammatory cytokines IL-1β, IL-6, and TNF-α. Moreover, nuclear expression of phospho-p65 was downregulated significantly in the SGF group compared with that in the Pb exposed group (**Figures 7C, D**). These results suggested that SGF treatment decreased activation of the NF-κB/IκBα/IKKα pathway induced by lead in the kidney.

In some inflammatory diseases, nuclear translocation of NF-κB p65 is represented as an increase in the nucleus:cytoplasm ratio. It has been reported that the nuclear translocation of p65 induces TNF-α transcription (Lee et al., 2011). To further demonstrate that pro-inflammatory cytokines induced activation of the NF-κB pathway, we measured nuclear translocation of NF-κB p65 in HEK-293 cells by confocal laser scanning microscope. SGF modulated nuclear translocation of NF-κB p65 significantly compared with that in Pb exposed HEK-293 cells (**Figures 7E, F**), which resulted in a significant decrease of NF-κB p65 in the nuclear fraction of cells. Also, the antioxidant and anti-inflammatory activities of SGF *in vivo* and *in vitro* provided important information. That is, SGF may prevent lead-induced nephrotoxicity by reducing ROS production, and then blocking the interconnection of ROS and NF-κB/IκB/IKK pathway.

## Conclusion

We demonstrated, for the first time, that SGF ameliorated lead-induced nephrotoxicity by reducing oxidative stress and inflammation *in vivo* and *in vitro*. The antioxidant mechanism of SGF was related to activation of the Keap1/Nrf2/HO-1 antioxidative pathway. The anti-inflammatory mechanism of SGF related to inhibition of ROS and pro-inflammatory cytokines triggered the NF-κB inflammatory pathway through blockade of inhibitors of IκB degradation, phosphorylation of NF-κB p65, and nuclear translocation of p65. Our findings suggest that SGF could be a natural antioxidant and anti-inflammatory agent for treating lead-induced nephrotoxicity in children.

## Data Availability Statement

The raw data supporting the conclusions of this article will be made available by the authors, without undue reservation, to any qualified researcher.

## Ethics Statement

The animal study was reviewed and approved by the Animal Ethics Committee of Zhejiang Chinese Medical University.

## Author Contributions

YS and DX contributed conception and design of the study. CT performed the animal experiments and data analysis. XY and XYZ performed the cell experiments. YS, CT, XY, YF, and XXZ collected and collated the data. DX wrote the first draft of the manuscript. All authors contributed to the article and approved the submitted version.

## Funding

This research project was funded by the National Natural Science Foundation of China (81374048, 81673656), the Zhejiang Provincial Natural Science Foundation, China (LY18H280002), and the Opening Project of Zhejiang Provincial Preponderant and Characteristic Subject of Key University (Traditional Chinese Pharmacology), Zhejiang Chinese Medical University (ZYAOXZD2019002).

## Conflict of Interest

The authors declare that the research was conducted in the absence of any commercial or financial relationships that could be construed as a potential conflict of interest.

## References

[B1] Adhikari A. Darbar S. Chatterjee T. Das M. Polley N. Bhattacharyya M. (2018). Spectroscopic studies on dual role of natural flavonoids in detoxification of lead poisoning: Bench-to-bedside preclinical trial. ACS Omega 3 (11), 15975–15987. 10.1021/acsomega.8b02046 30556021 PMC6288805

[B2] Aladaileh S. H. Khafaga A. F. Abd E. M. Al-Gabri N. A. Abukhalil M. H. Alfwuaires M. A. (2020). *Spirulina platensis* ameliorates the sub chronic toxicities of lead in rabbits via anti-oxidative, anti- inflammatory, and immune stimulatory properties. Sci. Total Environ. 701, 134879. 10.1016/j.scitotenv.2019.134879 31734488

[B3] Bao Y. Li H. Li Q. Y. Li Y. Li F. Zhang C. F. (2018). Therapeutic effects of *Smilax glabra* and *Bolbostemma paniculatum* on rheumatoid arthritis using a rat paw edema model. Biomed. Pharmacother. 108, 309–315. 10.1016/j.biopha.2018.09.004 30227323

[B4] Bonaterra G. A. Bronischewski K. Hunold P. Schwarzbach H. Heinrich E. U. Fink C. (2020). Anti-inflammatory and anti-oxidative effects of Phytohustil((R)) and root extract of *Althaea officinalis* L. on macrophages in vitro. Front. Pharmacol. 11, 290. 10.3389/fphar.2020.00290 32256361 PMC7090173

[B5] Bravo Y. Quiroz Y. Ferrebuz A. Vaziri N. D. Rodriguez-Iturbe B. (2007). Mycophenolate mofetil administration reduces renal inflammation, oxidative stress, and arterial pressure in rats with lead-induced hypertension. Am. J. Physiol. Renal Physiol. 293 (2), F616–F623. 10.1152/ajprenal.00507.2006 17567935

[B6] Cai Y. Tu J. Pan S. Jiang J. Shou Q. Ling Y. (2015). Medicinal effect and its JP2/RyR2-based mechanism of *Smilax glabra* flavonoids on angiotensin II-induced hypertrophy model of cardiomyocytes. J. Ethnopharmacol. 169, 435–440. 10.1016/j.jep.2015.04.026 25926285

[B7] Cao Y. Wang D. Li Q. Liu H. Jin C. Yang J. (2020). Activation of Nrf2 by lead sulfide nanoparticles induces impairment of learning and memory. Metallomics 12 (1), 34–41. 10.1039/c9mt00151d 31687725

[B8] Choy K. W. Murugan D. Leong X. F. Abas R. Alias A. Mustafa M. R. (2019). Flavonoids as natural anti-inflammatory agents targeting nuclear factor-kappa B (NFkappaB) signaling in cardiovascular diseases: A mini review. Front. Pharmacol. 10, 1295. 10.3389/fphar.2019.01295 31749703 PMC6842955

[B9] Gargouri M. Soussi A. Akrouti A. Magne C. El F. A. (2019). Potential protective effects of the edible alga *Arthrospira platensis* against lead-induced oxidative stress, anemia, kidney injury, and histopathological changes in adult rats. Appl. Physiol. Nutr. Metab. 44 (3), 271–281. 10.1139/apnm-2018-0428 30138569

[B10] Gargouri M. Akrouti A. Magné C. El Feki A. Soussi A. (2020). Protective effects of spirulina against hemato-biochemical alterations, nephrotoxicity, and DNA damage upon lead exposition. Hum. Exp. Toxicol. 39 (6), 855–869. 10.1177/0960327120903490 1538319085. 32003233

[B11] Gautam P. Flora S. J. S. (2010). Oral supplementation of gossypin during lead exposure protects alteration in heme synthesis pathway and brain oxidative stress in rats. Nutrition 26 (5), 563–570. 10.1016/j.nut.2009.06.008 19647414

[B12] Gupta S. C. Sharma A. Mishra M. Mishra R. K. Chowdhuri D. K. (2010). Heat shock proteins in toxicology: how close and how far? Life Sci. 86 (11-12), 377–384. 10.1016/j.lfs.2009.12.015 20060844

[B13] Harari F. Sallsten G. Christensson A. Petkovic M. Hedblad B. Forsgard N. (2018). Blood lead levels and decreased kidney function in a population-based cohort. Am. J. Kidney Dis. 72 (3), 381–389. 10.1053/j.ajkd.2018.02.358 29699886

[B14] Hou G. Surhio M. M. Ye H. Gao X. Ye Z. Li J. (2019). Protective effects of a *Lachnum* polysaccharide against liver and kidney injury induced by lead exposure in mice. Int. J. Biol. Macromol. 124, 716–723. 10.1016/j.ijbiomac.2018.11.133 30448488

[B15] Jiang S. Shang M. Mu K. Jiang N. Wen H. Wang R. (2018). In vitro and in vivo toxic effects and inflammatory responses induced by carboxylated black carbon-lead complex exposure. Ecotoxicol. Environ. Saf. 165, 484–494. 10.1016/j.ecoenv.2018.09.040 30219712

[B16] Lee S. T. Wong P. F. Cheah S. C. Mustafa M. R. (2011). Alpha-tomatine induces apoptosis and inhibits nuclear factor-kappa B activation on human prostatic adenocarcinoma PC-3 cells. PLoS One 6 (4), e18915. 10.1371/journal.pone.0018915 21541327 PMC3082542

[B17] Li Y. Darwish W. S. Chen Z. Hui T. Wu Y. Hirotaka S. (2019). Identification of lead-produced lipid hydroperoxides in human HepG2 cells and protection using rosmarinic and ascorbic acids with a reference to their regulatory roles on Nrf2-Keap1 antioxidant pathway. Chem. Biol. Interact. 314, 108847. 10.1016/j.cbi.2019.108847 31610155

[B18] Li D. X. Wang C. N. Wang Y. Ye C. L. Jiang L. Zhu X. Y. (2020). NLRP3 inflammasome-dependent pyroptosis and apoptosis in hippocampus neurons mediates depressive-like behavior in diabetic mice. Behav. Brain Res. 391, 112684. 10.1016/j.bbr.2020.112684 32454054

[B19] Liu C. M. Ma J. Q. Sun Y. Z. (2012a). Puerarin protects rat kidney from lead-induced apoptosis by modulating the PI3K/Akt/eNOS pathway. Toxicol. Appl. Pharm. 258 (3), 330–342. 10.1016/j.taap.2011.11.015 22172631

[B20] Liu C. M. Sun Y. Z. Sun J. M. Ma J. Q. Cheng C. (2012b). Protective role of quercetin against lead-induced inflammatory response in rat kidney through the ROS-mediated MAPKs and NF-kappaB pathway. Biochim. Biophys. Acta 1820 (10), 1693–1703. 10.1016/j.bbagen.2012.06.011 22728154

[B21] Lu C. L. Zhu W. Wang M. Xu X. J. Lu C. J. (2014). Antioxidant and anti-inflammatory activities of phenolic-enriched extracts of *Smilax glabra* . Evid. Based Complement. Alternat. Med. 2014, 910438. 10.1155/2014/910438 25477999 PMC4244943

[B22] Luo Q. Cai Z. Tu J. Ling Y. Wang D. Cai Y. (2019). Total flavonoids from *Smilax glabra* Roxb blocks epithelial-mesenchymal transition and inhibits renal interstitial fibrosis by targeting miR-21/PTEN signaling. J. Cell. Biochem. 120 (3), 3861–3873. 10.1002/jcb.27668 30304552

[B23] Mohamed R. S. Fouda K. Akl E. M. (2020). Hepatorenal protective effect of flaxseed protein isolate incorporated in lemon juice against lead toxicity in rats. Toxicol. Rep. 7, 30–35. 10.1016/j.toxrep.2019.12.001 31890606 PMC6926353

[B24] Nakano H. Nakajima A. Sakon-Komazawa S. Piao J. H. Xue X. Okumura K. (2006). Reactive oxygen species mediate crosstalk between NF-kappaB and JNK. Cell Death Differ. 13 (5), 730–737. 10.1038/sj.cdd.4401830 16341124

[B25] Nam S. M. Seo J. S. Go T. H. Nahm S. S. Chang B. J. (2019). Ascorbic acid supplementation prevents the detrimental effects of prenatal and postnatal lead exposure on the purkinje cell and related proteins in the cerebellum of developing rats. Biol. Trace Elem. Res. 190 (2), 446–456. 10.1007/s12011-018-1572-y 30488169

[B26] Navarro-Moreno L. G. Quintanar-Escorza M. A. González S. Mondragón R. Cerbón-Solorzáno J. Valdés J. (2009). Effects of lead intoxication on intercellular junctions and biochemical alterations of the renal proximal tubule cells. Toxicol. Vitro 23 (7), 1298–1304. 10.1016/j.tiv.2009.07.020 19619637

[B27] Oh B. M. Lee S. J. Park G. L. Hwang Y. S. Lim J. Park E. S. (2019). Erastin inhibits septic shock and inflammatory gene expression via suppression of the NF-kappaB pathway. J. Clin. Med. 8 (12), E2210. 10.3390/jcm8122210 31847346 PMC6947339

[B28] Oosterhuis N. R. Fernandes R. Maicas N. Bae S. E. Pombo J. Gremmels H. (2017). Extravascular renal denervation ameliorates juvenile hypertension and renal damage resulting from experimental hyperleptinemia in rats. J. Hypertens. 35 (12), 2537–2547. 10.1097/HJH.0000000000001472 28704264

[B29] Pastaci O. N. Duzgun E. D. Durmus S. Tuncdemir M. Uzun H. Gelisgen R. (2018). Selenium supplementation ameliorates electromagnetic field-induced oxidative stress in the HEK293 cells. J. Trace Elem. Med. Biol. 50, 572–579. 10.1016/j.jtemb.2018.04.008 29685784

[B30] Qu W. Du G. Feng B. Shao H. (2019). Effects of oxidative stress on blood pressure and electrocardiogram findings in workers with occupational exposure to lead. J. Int. Med. Res. 47 (6), 2461–2470. 10.1177/0300060519842446 31006320 PMC6567705

[B31] Rahmi E. P. Kumolosasi E. Jalil J. Husain K. Buang F. Abd R. A. (2020). Anti-hyperuricemic and anti-inflammatory effects of *Marantodes pumilum* as potential treatment for gout. Front. Pharmacol. 11, 289. 10.3389/fphar.2020.00289 32256360 PMC7092620

[B32] Ravipati E. S. Mahajan N. N. Sharma S. Hatware K. V. Patil K. (2019). The toxicological effects of lead and its analytical trends: an update from 2000 to 2018. Crit. Rev. Anal. Chem., 1–16. 10.1080/10408347.2019.1678381 31650860

[B33] Rozenberg K. Wollman A. Ben-Shachar M. Argaev-Frenkel L. Rosenzweig T. (2019). Anti-inflammatory effects of *Sarcopoterium spinosum* extract. J. Ethnopharmacol. 112391. 10.1016/j.jep.2019.112391 31730890

[B34] Salama S. A. Arab H. H. Maghrabi I. A. Hassan M. H. AlSaeed M. S. (2016). Gamma-glutamyl cysteine attenuates tissue damage and enhances tissue regeneration in a rat model of lead-induced nephrotoxicity. Biol. Trace Elem. Res. 173 (1), 96–107. 10.1007/s12011-016-0624-4 26767370

[B35] Sas K. Szabo E. Vecsei L. (2018). Mitochondria, oxidative stress and the kynurenine system, with a focus on ageing and neuroprotection. Molecules 23 (1), E191. 10.3390/molecules23010191 29342113 PMC6017505

[B36] Shenai-Tirodkar P. S. Gauns M. U. Mujawar M. Ansari Z. A. (2017). Antioxidant responses in gills and digestive gland of oyster *Crassostrea madrasensis* (Preston) under lead exposure. Ecotoxicol. Environ. Saf. 142, 87–94. 10.1016/j.ecoenv.2017.03.056 28391094

[B37] Shirpoor A. Rezaei F. Fard A. A. Afshari A. T. Gharalari F. H. Rasmi Y. (2016). Ginger extract protects rat’s kidneys against oxidative damage after chronic ethanol administration. Biomed. Pharmacother. 84, 698–704. 10.1016/j.biopha.2016.09.097 27710894

[B38] Sudjarwo S. A. Eraiko K. Sudjarwo G. W. Koerniasari (2019). The potency of chitosan-Pinus merkusii extract nanoparticle as the antioxidant and anti-caspase 3 on lead acetate-induced nephrotoxicity in rat. J. Adv. Pharm. Technol. Res. 10 (1), 27–32. 10.4103/japtr.JAPTR_306_18 30815385 PMC6383345

[B39] Turksoy V. A. Tutkun L. Iritas S. B. Gunduzoz M. Deniz S. (2019). The effects of occupational lead exposure on selected inflammatory biomarkers. Arh. Hig. Rada Toksikol. 70 (1), 36–41. 10.2478/aiht-2019-70-3214 30956219

[B40] Upadhyaya B. Liu Y. Dey M. (2019). Phenethyl isothiocyanate exposure promotes oxidative stress and suppresses Sp1 transcription factor in cancer stem cells. Int. J. Mol. Sci. 20 (5), E1027. 10.3390/ijms20051027 30818757 PMC6429440

[B41] Vicente-Vicente L. Gonzalez-Calle D. Casanova A. G. Hernandez-Sanchez M. T. Prieto M. Rama-Merchan J. C. (2019). Quercetin, a promising clinical candidate for the prevention of contrast-induced nephropathy. Int. J. Mol. Sci. 20 (19), 4961. 10.3390/ijms20194961 PMC6801677 31597315

[B42] Wang L. Lin S. Li Z. Yang D. Wang Z. (2013). Protective effects of puerarin on experimental chronic lead nephrotoxicity in immature female rats. Hum. Exp. Toxicol. 32 (2), 172–185. 10.1177/0960327112462729 23315276

[B43] Wang Q. Zhao L. Zhao H. Liu X. Gao L. Cheng N. (2019). Complexation of luteolin with lead (II): Spectroscopy characterization and theoretical researches. J. Inorg. Biochem. 193, 25–30. 10.1016/j.jinorgbio.2019.01.007 30669063

[B44] Wang S. W. Fang Y. J. Yu X. F. Guo L. Zhang X. X. Xia D. Z. (2019). The flavonoid-rich fraction from rhizomes of *Smilax glabra* Roxb. ameliorates renal oxidative stress and inflammation in uric acid nephropathy rats through promoting uric acid excretion. Biomed. Pharmacother. 111, 162–168. 10.1016/j.biopha.2018.12.050 30579255

[B45] Wang Q. Zhao H. Zhu M. Gao L. Cheng N. Cao W. (2020). Spectroscopy characterization, theoretical study and antioxidant activities of the flavonoids-Pb(II) complexes. J. Mol. Struct. 1209, 127919. 10.1016/j.molstruc.2020.127919

[B46] Xia D. Z. Yu X. F. Liao S. P. Shao Q. J. Mou H. L. Ma W. (2010). Protective effect of *Smilax glabra* extract against lead-induced oxidative stress in rats. J. Ethnopharmacol. 130 (2), 414–420. 10.1016/j.jep.2010.05.025 20580805

[B47] Xia D. Z. Fan Y. S. Zhang P. H. Fu Y. Ju M. T. Zhang X. S. (2013). Protective effects of the flavonoid-rich fraction from rhizomes of *Smilax glabra* Roxb. on carbon tetrachloride-induced hepatotoxicity in rats. J. Membrane Biol. 246 (6), 479–485. 10.1007/s00232-013-9560-9 23681353

[B48] Xiao J. Chen B. Wang Q. Yang L. Guo H. (2018). Paeonin extracted from potatoes protects gastric epithelial cells from H2O2-induced oxidative damage in vitro by PI3K/Akt-mediated Nrf2 signaling pathway. Sci. Rep. 8 (1), 10865. 10.1038/s41598-018-28772-5 30022028 PMC6052145

[B49] Yabe J. Nakayama S. M. Nakata H. Toyomaki H. Yohannes Y. B. Muzandu K. (2020). Current trends of blood lead levels, distribution patterns and exposure variations among household members in Kabwe, Zambia. Chemosphere 243, 125412. 10.1016/j.chemosphere.2019.125412 31995873

[B50] Yamawaki K. Kanda H. Shimazaki R. (2018). Nrf2 activator for the treatment of kidney diseases. Toxicol. Appl. Pharmacol. 360, 30–37. 10.1016/j.taap.2018.09.030 30248418

[B51] Yedjou C. G. Tchounwou H. M. Tchounwou P. B. (2015). DNA damage, cell cycle arrest, and apoptosis induction caused by lead in human leukemia cells. Int. J. Env. Res. Pub. He. 13 (1), h13010056. 10.3390/ijerph13010056 PMC4730447 26703663

[B52] Zhao H. Yan L. Xu X. Jiang C. Shi J. Zhang Y. (2018). Potential of *Bacillus subtilis* lipopeptides in anti-cancer I: induction of apoptosis and paraptosis and inhibition of autophagy in K562 cells. AMB Express 8 (1), 78. 10.1186/s13568-018-0606-3 29777449 PMC5959823

[B53] Zhao J. W. Zheng C. Y. Wei H. Wang D. W. Zhu W. (2018). Proapoptic and immunotoxic effects of sulfur-fumigated polysaccharides from *Smilax glabra* Roxb. in RAW264.7cells. Chem. Biol. Interact. 292, 84–93. 10.1016/j.cbi.2018.07.009 30012344

