# Supplementary material for: Protective Effects of Smilax glabra Roxb. Against Lead-Induced Renal Oxidative Stress, Inflammation and Apoptosis in Weaning Rats and HEK-293 Cells
Source: Front Pharmacol. 2020 Sep 2;11:556248. doi: 10.3389/fphar.2020.556248 (PMC7493636; doi:10.3389/fphar.2020.556248)
Supplement: Supplementary file 1 [file DataSheet_1.pdf]

Supplement 1. Total flavonoids determination and quantitative analysis of five flavonoids compounds in *Smilax glabra* flavonoids extract (SGF)

Total Flavonoids Determination of SGF

The total flavonoid content of SGF was measured using a colorimetric assay. A known volume (1 mL) of the extract or standard solution of rutin was added to a 10 mL volumetric flask. Distilled water was added to make a volume of 5 mL. Firstly, 0.3 mL of $NaNO_2$ (5%, w/v) was added to the flask. After 5 min, 0.6 mL of $AlCl_3$ (10%, w/v) was added, and after 6 min, 2 mL of NaOH (1.0 M) was added to the mixture followed by the addition of 2.1 mL of distilled water. Absorbance was read at 510 nm against the blank (water), and total flavonoids content is expressed as milligrams of rutin equivalents per g of SGF. The standard curve of rutin showed in **Figure S1**.

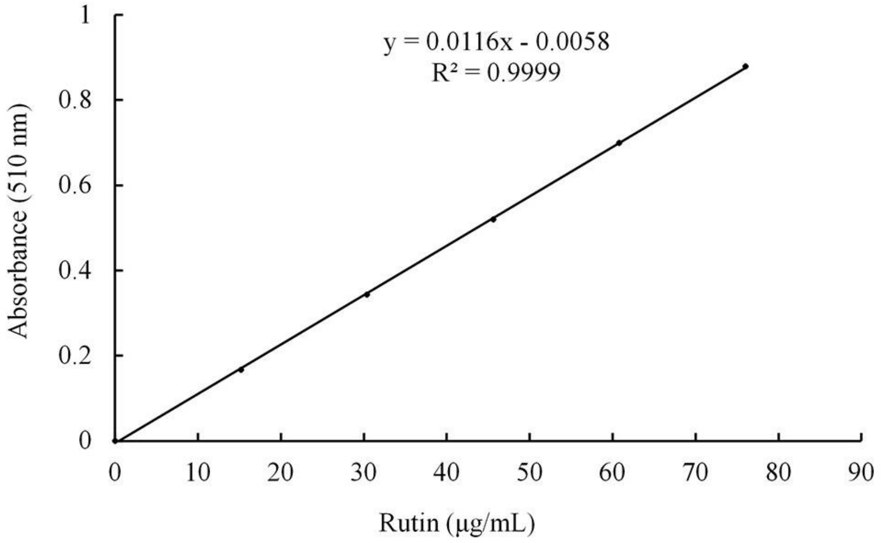

Figure S1 Standard curve of rutin.

Ultra Performance Liquid Chromatograph analysis of SGF

The quantitative analysis of five flavonoids compounds in SGF was performed with UPLC (ACQUITY UPLC H-Class, Waters, USA). The SGF freeze-dried powder was dissolved in methanol and the concentration was 82.72 μg/mL. Then the methanolic solution of SGF was filtered through 0.22 μm membrane filter (Nylon) for further analysis. An acquity UPLC C_{18} column (50 mm × 2.1 mm; 1.7 μm, Waters) was used for separating the flavonoids compounds. The column temperature was 35 °C and the injection volume was 2 μL. The mobile phase consisted of A (100% acetonitrile) and B (0.3% formic acid in water) with a flow rate of 0.35 mL/min. An isocratic elution was

performed with 10 min, and the mobile phase ratio is 14.8% A and 85.2% B. Before injecting the next sample, the gradient was maintained as such for 10 min. Detection wavelengths were 290 nm for flavonoids compounds. The content of flavonoids compounds in rhizomes of SGF was calculated from the external calibration curves of standards (**Figure S2, Table S1**).

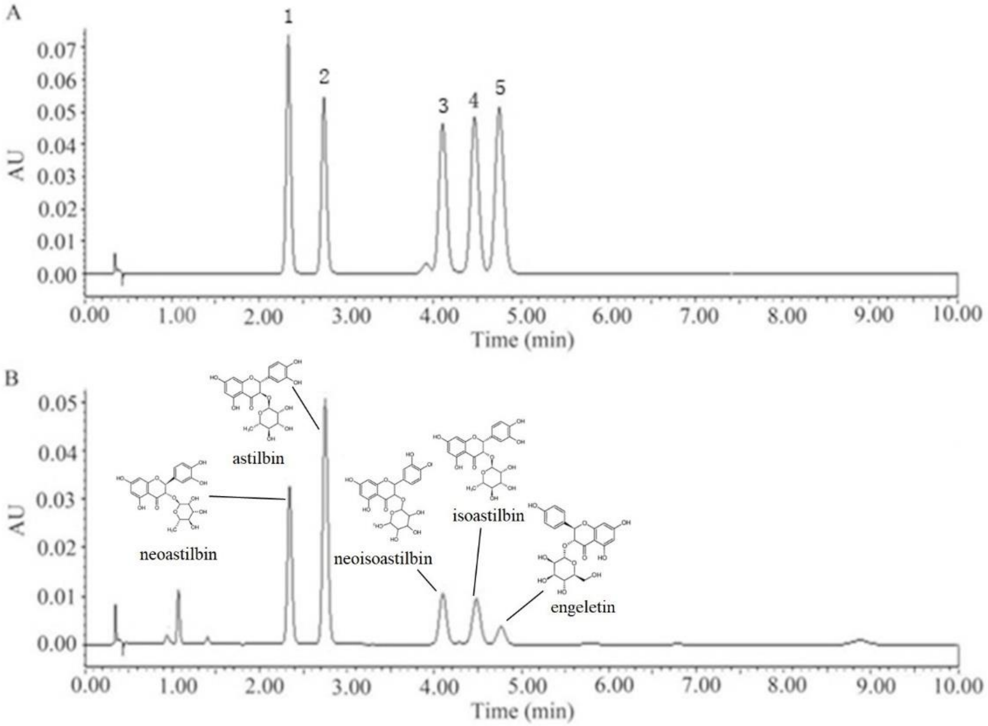

Figure S2 UPLC analysis of SGF. **(A)** Five flavonoids compounds mixed standards (1-neoastilbin, 2-astilbin, 3-neoisoastilbin, 4-isoastilbin, 5-engeletin); **(B)** UPLC analysis of SGF.

Table S1 The linear relationship of five flavonoids compounds of SGF.

Compound	Regression equation	R^2	Linear range (μg/mL)
Neoastilbin	y = 12854x - 9.833	0.9993	0.0859-36.67
Astilbin	y = 13604x + 1490.2	0.9995	0.0781-33.33
Neoisoastilbin	y = 12854x - 9.833	0.9999	0.156-10
Isoastilbin	y = 15200x + 30.46	0.9994	0.1953-10.41
Engeletin	y = 12234x - 385.48	0.9999	0.1875-24
