# Supplementary material for: Protective Effects of Smilax glabra Roxb. Against Lead-Induced Renal Oxidative Stress, Inflammation and Apoptosis in Weaning Rats and HEK-293 Cells
Source: Front Pharmacol. 2020 Sep 2;11:556248. doi: 10.3389/fphar.2020.556248 (PMC7493636; doi:10.3389/fphar.2020.556248)

Supplement 2. Full Length Images of the Blots

According to the reviewer's suggestion, we adjusted our protocol of the western blot, and determined the protein expression of p65 and phospho-p65 in cytoplasmic fraction and phospho-p65 in nuclear fraction. We provided the original images with molecular weight markers of these three blots in the Supplementary Material 2. However, in our previous protocol of the western blot, after SDS-polyacrylamide gel electrophoresis, the gel was cut into small strips according to protein molecular weight, then transferred onto the corresponding size of the PVDF membranes. Meanwhile, we separated the marker from the gel of the target proteins. Thus, we can only provide the individual original image of the target protein instead of the full length images of blots. We sincerely hope that the reviewer can understand.

Full Length Images of the Blots of p65 and Phospho-p65 in Cytoplasmic Fraction of HEK-293 Cells (Figure 7C)

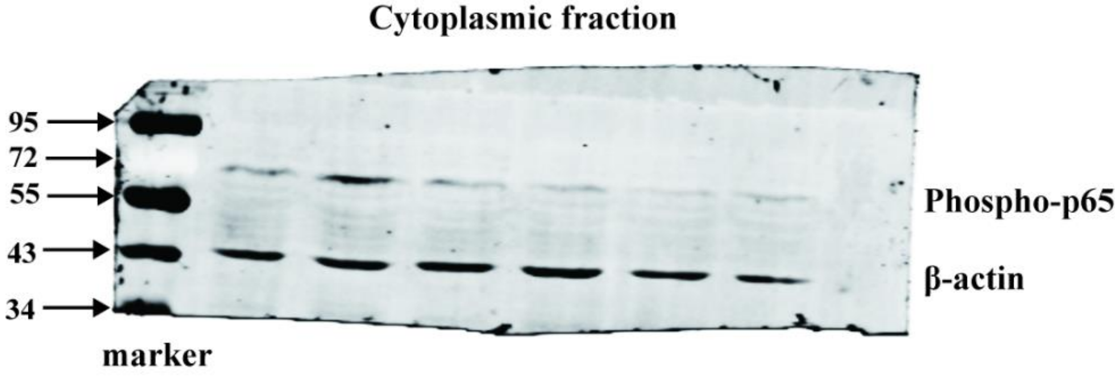

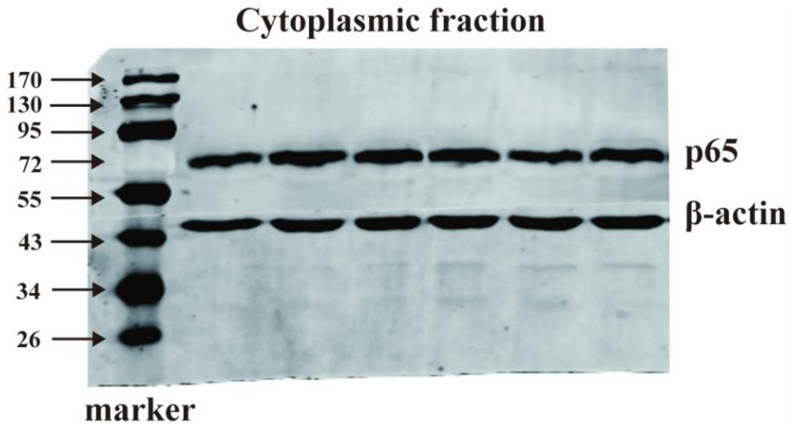

Full Length Image of the Blot of Phospho-p65 in Nuclear Fraction of HEK-293 Cells (Figure 7C)

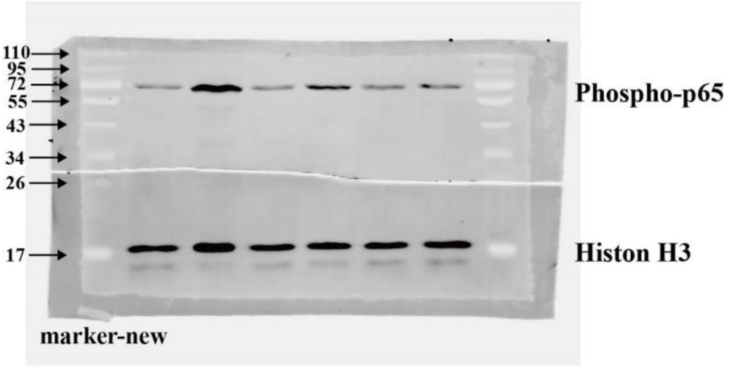

Original Images of the Blots of Full-caspase-1 and Cleaved-caspase-1 (Figure 5E)

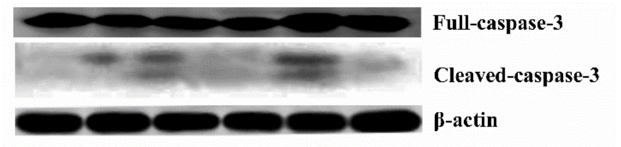

Original Images of the Blots of Keap1, Nrf2 and HO-1 (Figure 6H)

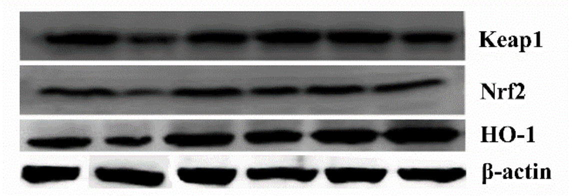

Original Images of the Blots of p65, Phospho-p65, IκBα, Phospho-p65, IKKα and Phospho-IKKα (Figure 7A)

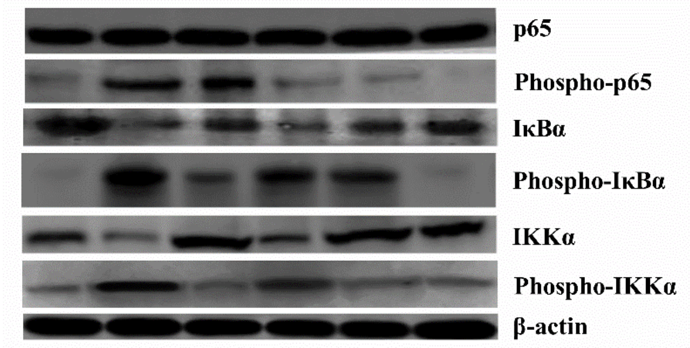

Supplement: Supplementary file 2 [file DataSheet_2.pdf]
